# Influence of phase correction of late gadolinium enhancement images on scar signal quantification in patients with ischemic and non-ischemic cardiomyopathy

**DOI:** 10.1186/s12968-015-0163-8

**Published:** 2015-08-07

**Authors:** John Stirrat, Sebastien Xavier Joncas, Michael Salerno, Maria Drangova, James White

**Affiliations:** Robarts Research Institute, University of Western Ontario, London, Ontario Canada; Division of Cardiology, Department of Medicine, University of Calgary, Calgary, Alberta Canada; Departments of Medicine, Radiology, and Biomedical Engineering, University of Virginia, Charlottesville, VA USA; Stephenson Cardiac Imaging Centre, Libin Cardiovascular Institute, University of Calgary, Calgary, Alberta Canada

**Keywords:** Cardiovascular magnetic resonance, Late gadolinium enhancement, Scar quantification, Ischemic cardiomyopathy, Non-ischemic cardiomyopathy, Phase-sensitive inversion recovery (PSIR)

## Abstract

**Background:**

Myocardial fibrosis imaging using late gadolinium enhancement (LGE) cardiac magnetic resonance (CMR) has been validated as a quantitative predictive marker for response to medical, surgical, and device therapy. To date, all such studies have examined conventional, non-phase corrected magnitude images.  However, contemporary practice has rapdily adopted phase-corrected image reconstruction. We sought to investigate the existence of any systematic bias between threshold-based scar quantification performed on conventional magnitude inversion recovery (MIR) and matched phase sensitive inversion recovery (PSIR) images.

**Methods:**

In 80 patients with confirmed ischemic (*N* = 40), or non-ischemic (*n* = 40) myocardial fibrosis, and also in a healthy control cohort (*N* = 40) without fibrosis, myocardial late enhancement was quantified using a Signal Threshold Versus Reference Myocardium technique (STRM) at ≥2, ≥3, and ≥5 SD threshold, and also using the Full Width at Half Maximal (FWHM) technique. This was performed on both MIR and PSIR images and values compared using linear regression and Bland-Altman analyses.

**Results:**

Linear regression analysis demonstrated excellent correlation for scar volumes between MIR and PSIR images at all three STRM signal thresholds for the ischemic (*N* = 40, r = 0.96, 0.95, 0.88 at 2, 3, and 5 SD, *p* < 0.0001 for all regressions), and non ischemic (*N* = 40, r = 0.86, 0.89, 0.90 at 2, 3, and 5 SD, *p* < 0.0001 for all regressions) cohorts. FWHM analysis demonstrated good correlation in the ischemic population (*N* = 40, r = 0.83, *p* < 0.0001). Bland-Altman analysis demonstrated a systematic bias with MIR images showing higher values than PSIR for ischemic (3.3 %, 3.9 % and 4.9 % at 2, 3, and 5 SD, respectively), and non-ischemic (9.7 %, 7.4 % and 4.1 % at ≥2, ≥3, and ≥5 SD thresholds, respectively) cohorts. Background myocardial signal measured in the control population demonstrated a similar bias of 4.4 %, 2.6 % and 0.7 % of the LV volume at 2, 3 and 5 SD thresholds, respectively. The bias observed using FWHM analysis was −6.9 %.

**Conclusions:**

Scar quantification using phase corrected (PSIR) images achieves values highly correlated to those obtained on non-corrected (MIR) images. However, a systematic bias exists that appears exaggerated in non-ischemic cohorts. Such bias should be considered when comparing or translating knowledge between MIR- and PSIR-based imaging.

## Background

**Fig. 1:**
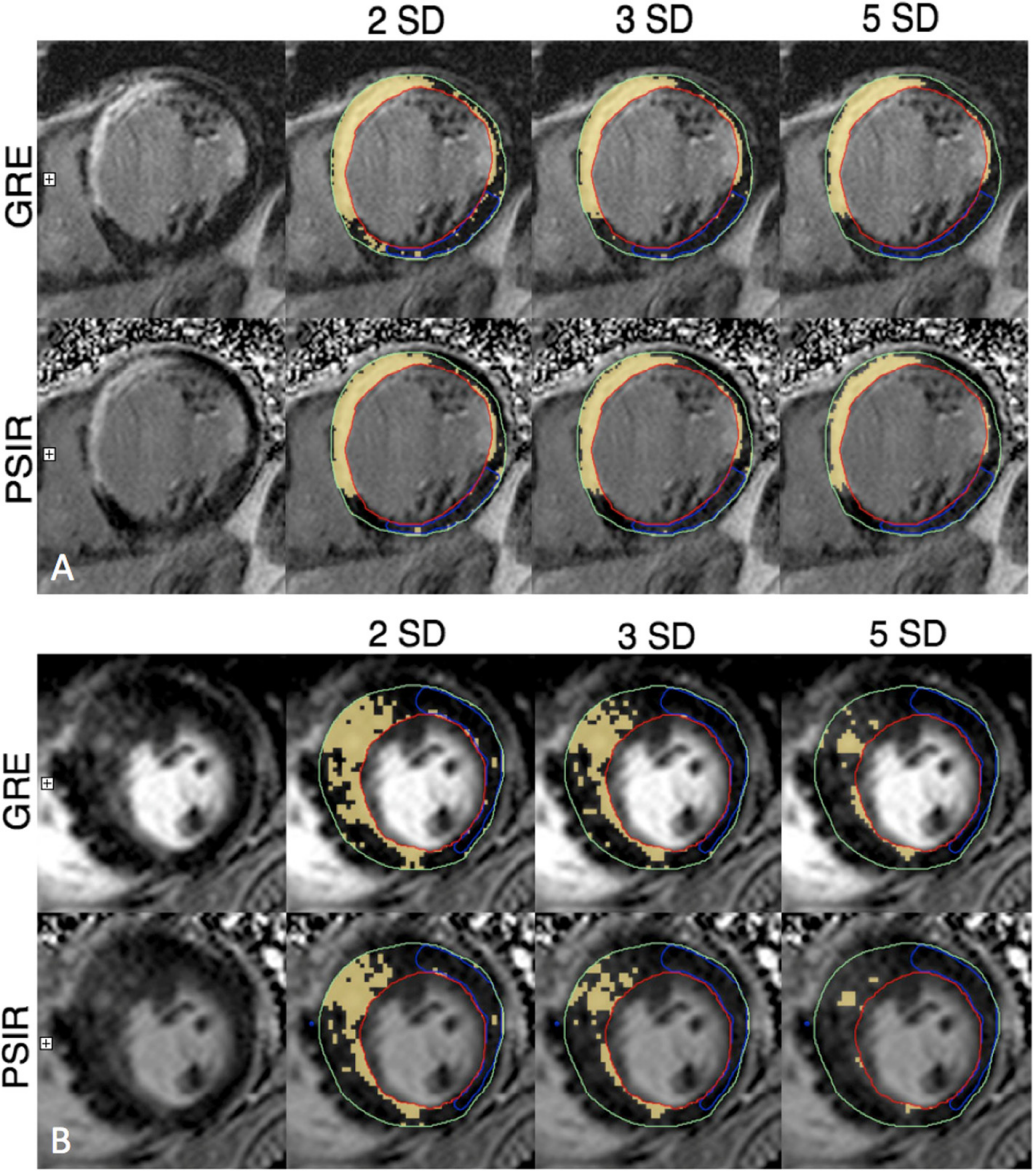
Example of quantitative analysis of late enhancement in a patient with **a** ischemic cardiomyopathy and **b** non-ischemic cardiomyopathy. Optimally nulled MIR image (top panel), and matched PSIR image (lower panel) are shown with scar (yellow) identified at 2, 3, and 5 SD thresholds above the mean signal intensity of normal reference myocardium (shown in blue)

Late gadolinium enhancement cardiovascular magnetic resonance (LGE-CMR) is a well-established clinical tool for the assessment of irreversible myocardial injury or “scar” in patients with ischemic and non-ischemic cardiomyopathy [1, 2]. Several studies now suggest strong prognostic utility for the quantification of ischemic scar signal to predict response to medical [3], surgical [4, 5] and device therapy [6–8]. Furthermore, in those with dilated cardiomyopathy (DCM) [9–11] and hypertrophic cardiomyopathy (HCM) [12] the presence and extent of non-ischemic LGE appears to predict future cardiovascular events. These studies have employed signal-threshold based approaches to segment bright scar signal from the otherwise dark or “nulled” myocardium, this signal gradient effectively provided by magnitude-reconstruction inversion recovery (MIR) imaging, as was originally described by Simonetti, et al. [13]. However, due to this technique’s inherent dependence on accurate prescription of the inversion time (TI) to optimally “null” normal myocardium [14], an alternative approach called phase-sensitive inversion recovery (PSIR) has been introduced [15]. This technique exploits collection of a reference phase image on alternating heartbeats which enables restoration of the voxel polarity using a phase-sensitive reconstruction to correct tissue signal intensities related to inaccurate TI prescription (Fig. 1). By eliminating a need for accurate TI prescription this technique has realized rapid and widespread clinical adoption.

While early validation studies compared basic scar area measures from PSIR and MIR images (predominantly by visual planimetry) [15–19], the current paradigm of scar signal quantification has not been appropriately evaluated. Specifically, the effect of PSIR reconstruction on the behavior of scar volumetry obtained using multi-threshold Signal Threshold versus Reference Myocardium (STRM) and Full Width Half of Maximum (FWHM) techniques is unknown. Given the marked alteration in the range of signal intensities with phase-sensitive reconstruction [15] it cannot be assumed that such assessments would be unaffected. If such differences exist, their definition would be of importance for the design and interpretation of multi-center clinical trials or registries that include heterogeneous site adoption of the PSIR technique.

The aim of this study was to systematically compare scar volume measurements acquired by currently published signal-threshold techniques obtained from spatially and temporally matched MIR and PSIR LGE images in patients with ischemic and non-ischemic myocardial scar.

## Methods

### Patient population

A total of 80 consecutive patients with definite myocardial scar were identified from initial short-axis, free breathing (single shot) LGE imaging, 40 with ischemic and 40 with non-ischemic myocardial scar. These patients underwent a standardized, TI-optimized LGE imaging protocol [12, 13] to obtain spatially and temporally matched PSIR and MIR reconstructed images. In addition, a control cohort of 40 patients with no identifiable scar was identified to evaluate the effects of PSIR versus MIR on normal tissue signal and background image noise. This cohort of patients were all referred for the exclusion of structural heart disease but determined to have a normal CMR study.

All patients provided written informed consent, and the study protocol was approved by the Institutional Health Research Ethics Board. Patients with an estimated glomerular filtration rate (eGFR) ≤ 30 mL/min/m^2^ were excluded from this study.

### CMR protocol

Patients were scanned using a 3.0 Tesla scanner (TIM TRIO or Verio; Siemens Medical Systems, Germany) using a 32-channel phased-array radiofrequency coil. Cine functional imaging was performed in a standard fashion using an SSFP-based pulse sequence (TrueFISP) in sequential short-axis slices from the atrioventricular annulus to the left ventricular apex at 10 mm intervals (slice thickness 6 mm, gap 4 mm, TE 1.5 msec, TR 35–45 msec). At 5–7 min following the administration of 0.2 mmol/kg of gadolinium (Magnevist®, or Gadovist®, Bayer Inc. Canada) a free-breathing series of short-axis images were obtained using an SSFP-based “single shot inversion recovery” pulse sequence (slice thickness 8 mm, gap 2 mm, matrix 192 × 192, TE 1.2 msec, TR 960 msec, iPAT 2). This allowed for identification of obvious myocardial scar. At 10 min following contrast breath-held LGE imaging was performed in slice orientations identical to cine imaging using a segmented PSIR turbo-FLASH pulse sequence with meticulous attention given to prescribe the TI time to optimally null normal myocardium, as previously described [14] (matrix 256 × 192, slice thickness 6 mm, and gap 4 mm, TE 1.4 msec, TR 760 msec, iPAT 2). Both magnitude and phase-sensitive reconstruction of each LGE image were generated, ensuring that images were both temporally and spatially matched.

### CMR image analysis

Cine image datasets (*N* = 120) were de-identified and underwent blinded assessment of left ventricular (LV) end-diastolic and end-systolic volumes using commercially available, semi-automated software (CVI42, Circle Cardiovascular Inc., Calgary, Canada).

Using the same commercial software MIR and PSIR LGE image datasets were separately analyzed in random order. A single set of endocardial and epicardial contours were manually traced from the MIR dataset and then stored for use for all scar image analyses. This was done to ensure that signal volume differences were not introduced from human reproducibility errors. For STRM-based analyses (all patients) a large contiguous region of normal (“nulled”) myocardium was identified (30-50 % of total myocardial area whenever possible) for each slice, as shown in Fig. 1, and served as the reference for scar segmentation where scar signal is defined using a selected number of standard deviations (SD) above the mean reference signal [20, 21]. For this study we evaluated the most commonly reported ≥2, ≥3 and ≥5SD thresholds. The FWHM technique was incrementally evaluated in the ischemic sub-group (*N* = 40) with the densest region of scar (when present) traced for each slice. This served as a reference where scar was defined as any voxel with signal ≥50 % of the highest reference region signal [20]. The FWHM technique was not applied to the non-ischemic cohort as it included patients with dilated cardiomyopathy for which this technique may not be appropriate [22]. Total scar volume was expressed as a percent of the LV myocardial volume, and was obtained by dividing the summed scar volumes by the summed myocardial volumes (epicardium minus endocardium) for all analyzed slices.

Due to differences in signal intensity patterns between matched MIR and PSIR images, FWHM analysis of PSIR images required the signal intensity (SI) be normalized to the minimum SI of the myocardium in the PSIR image. This normalization was required since the signal intensity of normal myocardium on MIR images is near 0 (i.e. nulled), whereas in the PSIR images, which have twice the dynamic range, the myocardial signal is scaled near the center of the total dynamic range (approximately 1800 for our PSIR sequence).

### Statistical analysis

Continuous variables are reported as the mean ± SD, categorical variables as percentage of total. The correlation between MIR- and PSIR-based scar signal quantification as a percent of total LV volume was evaluated according to the Pearson correlation coefficient (r). Bland-Altman analysis was performed for all comparisons with bias estimated by the mean difference. Statistical analyses were performed using a commercially available software program (GraphPad Prism, Version 5.0, CA, USA). All probability values were 2-sided, and a *p*-value ≤ 0.05 was considered significant.

## Results

### Patient characteristics

Baseline patient characteristics are shown in Table 1. The mean age of the population was 57.9 ± 16.9 years. The mean ejection fraction (EF) of ischemic, non-ischemic and control groups were 35.8 ± 15.9 %, 56.3 ± 20.8 %, and 63.6 ± 15.4 %, respectively. Of patients with ischemic scar, 29 (72 %) had single coronary territory infarction and 11 (28 %) had ≥2 territories involved. Among patients with non-ischemic scar, 24 (60 %) had DCM with typical mid-wall linear scar, 14 (35 %) had HCM with patchy mid-wall scar and 2 patients (5 %) had a history congenital heart disease with mid-wall fibrosis at the right ventricular insertion sites.

**Table 1:** Baseline patient characteristics for each subgroup

	Ischemic	Non-Ischemic	Control
	*n* = 40	*n* = 40	*n* = 40
Patient Characteristics			
Age	62.8 ± 15.6	58.2 ± 16.5	52.6 ± 15.2
Female	9 (22.5 %)	3 (7.5 %)	12 (30.0 %)
Hypertension	19 (47.5 %)	16 (40.0 %)	15 (37.5 %)
Diabetes	7 (17.5 %)	4 (10.0 %)	3 (7.5 %)
Hyperlipidemia	20 (50.0 %)	14 (35.0 %)	11 (27.5 %)
Prior MI	40 (100.0 %)	0 (0.0 %)	0 (0.0 %)
Prior Revascularization	20 (50 %)	3 (7.5 %)	4 (10 %)
MRI Parameters			
LV EDV (mL ± SD)	215.4 ± 71.9	176.7 ± 86.4	160.4 ± 62.4
LV ESV (mL ± SD)	145.0 ± 70.0	91.3 ± 83.4	61.8 ± 46.5
LV EF (% ± SD)	35.8 ± 15.9	56.3 ± 20.8	63.6 ± 15.4
LV Mass (g ± SD)	171.3 ± 51.2	174.3 ± 69.1	145.8 ± 49.3

*MI* myocardial infarction; *LV* left ventricle; *EDV* end diastolic volume; *ESV* end systolic volume; *EF* ejection fraction

### Global myocardial scar burden

Overall, quantitative signal analysis of total myocardial scar volume identified significantly higher values for those with ischemic versus non-ischemic injury (Table 2). For example, by STRM-based analysis (≥5SD) of conventional MIR images the mean total scar volume of ischemic and non-ischemic cohorts was 21.3 % ± 13.0 % and 7.1 ± 7.7 %, respectively (p < 0.01).

**Table 2:** Mean late enhancement (±SD) expressed as percent of total LV volume for each subgroup as measured from optimized magnitude inversion recovery (MIR) and phase sensitive inversion recovery (PSIR) images

	Ischemic	Non-ischemic	Control
2SD			
MIR	37.86 ± 13.64*	27.53 ± 10.42*	13.55 ± 5.55*
PSIR	34.52 ± 13.64	18.21 ± 10.26	9.20 ± 3.72
3SD			
MIR	30.08 ± 14.05*	16.58 ± 9.68*	6.06 ± 3.11*
PSIR	26.14 ± 12.46	9.16 ± 8.70	3.44 ± 1.99
5SD			
MIR	21.27 ± 12.95*	7.05 ± 7.66*	1.32 ± 0.90*
PSIR	16.35 ± 10.05	2.97 ± 5.69	0.65 ± 0.68
FWHM			
MIR	15.41 ± 9.87*		
PSIR	22.35 ± 14.71		

*SD* standard deviation; *LV* left ventricle; *MIR* magnitude inversion recovery; *PSIR* phase sensitive inversion recovery; *FWHM* full-width at half-maximum

*p < 0.0001 for comparison between MIR and PSIR by paired-sample *t*-test at the respective threshold

Within the control population a measurable native myocardial signal was identified at all signal thresholds, supporting that background myocardial signal indeed contributes to total scar volume quantification using a regional tissue reference for application of STRM-based techniques. As shown in Table 2, this appeared modest at the ≥5SD signal threshold (1.3 ± 0.9 %), but increased in both absolute and relative contribution as signal threshold was lowered (6.1 ± 3.1 % at ≥3SD and 13.6 ± 5.6 % at ≥2SD).

### PSIR versus MIR scar volumes – STRM-based analysis

All study subjects with myocardial scar (*N* = 80) underwent multi-threshold STRM-based scar analysis. A strong correlation between PSIR and MIR derived measures of total scar volume was identified for all 3 signal thresholds. Correlation coefficients for ≥2SD, ≥3SD and ≥5SD thresholds were 0.93, 0.94, and 0.92, respectively. Bland-Altman analysis revealed a systematic bias for PSIR derived measures consistently falling below MIR derived measurements at all 3 thresholds. A mean absolute reduction in total percent scar volume of 6.5 %, 5.7 % and 4.5 % was identified at ≥2SD, ≥3SD and ≥5SD thresholds, respectively.

**Fig. 2:**
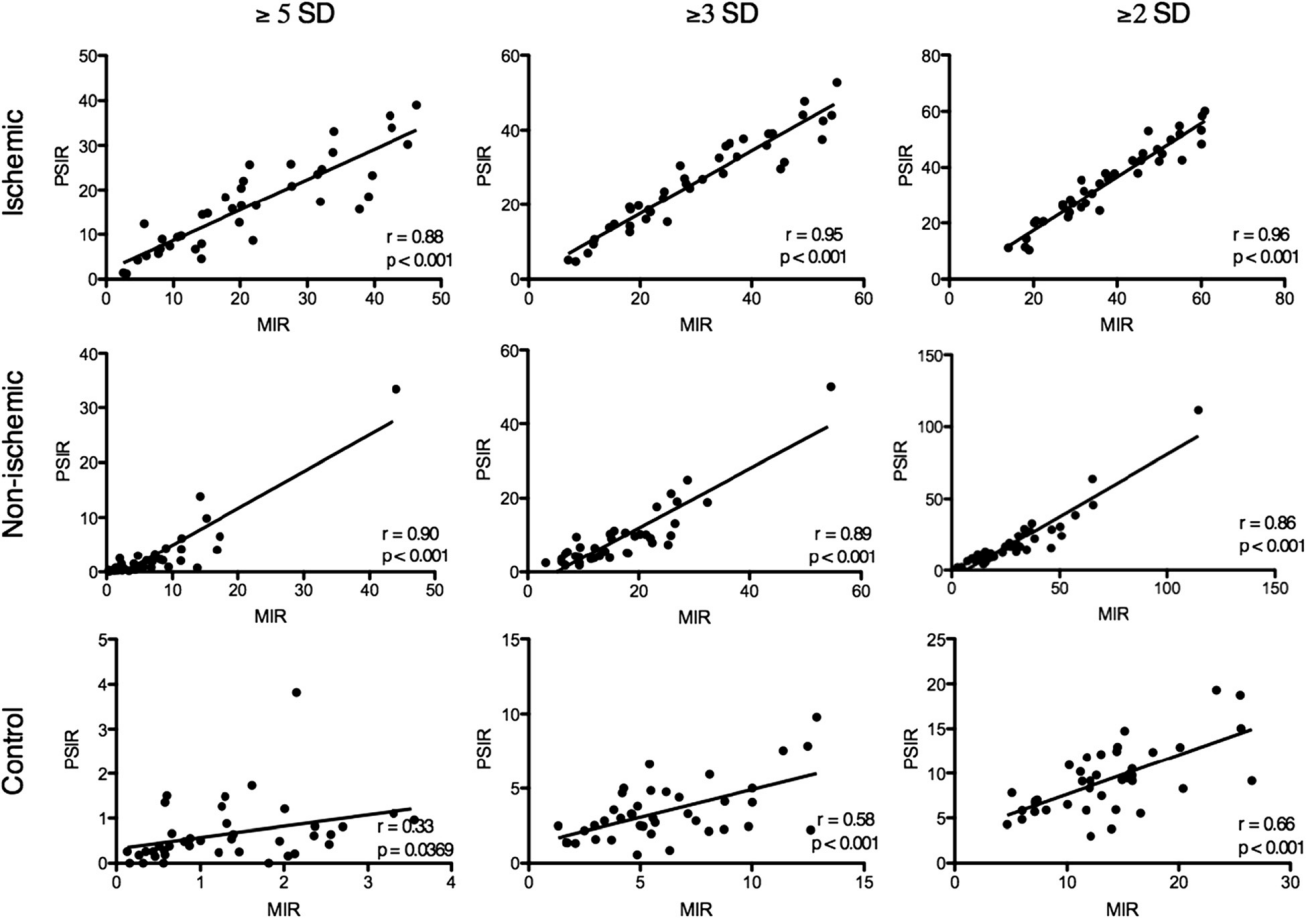
Linear regression analysis comparing myocardial late enhancement volume using MIR and PSIR for STRM-based analysis. Late enhancement is reported as percent of total LV myocardial volume at 2, 3, and 5 SD from the mean signal intensity of normal reference myocardium

**Fig. 3:**
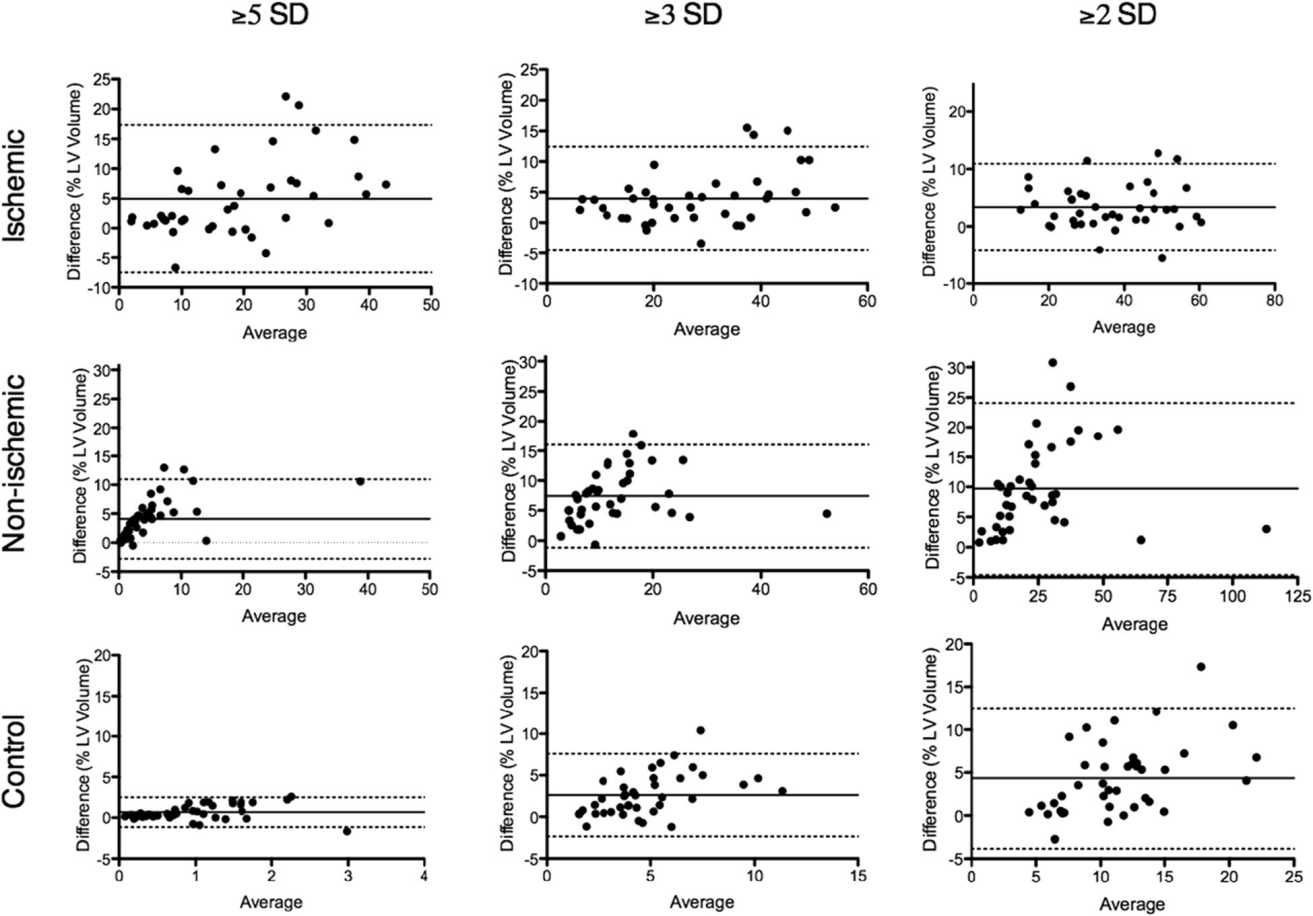
Bland-Altman analysis comparing myocardial late enhancement volume using MIR and PSIR for STRM-based analysis. Values indicate the mean difference of MIR-based scar volume quantification relative to PSIR. Late enhancement is reported as percent of total LV myocardial volume at ≥2, ≥3, and ≥5 SD from the mean signal intensity of normal reference myocardium. Long dashed lines represent bias, dotted lines represent 95 % confidence intervals

Similar analysis was performed following stratification for scar etiology. In those with ischemic scar, excellent correlation was seen between PSIR and MIR based measures at ≥2SD, ≥3SD and ≥5SD, respective correlation coefficients being 0.96, 0.95, and 0.88 (*p* < 0.0001 for all regressions) (Fig. 2). The mean bias at these thresholds was 3.3 %, 3.9 %, and 4.9 % (lower for PSIR) (Fig. 3), suggesting no association with the signal threshold selected (*p* = 0.36 by ANOVA). By comparison, those with non-ischemic scar showed lower correlation coefficients between PSIR and MIR based analysis (0.86, 0.89, and 0.90, respectively, p < 0.0001 for all regression) (Fig. 2). Although similarly showing a negative bias for PSIR based scar volumes, an inverse relationship of severity to the signal threshold selected was evident (9.7 %, 7.4 % and 4.1 % at ≥2, ≥3, and ≥5 SD thresholds, respectively, *p* < 0.001, by ANOVA. A summary of these biases is provided in Table 3 with respective Bland-Altman plots shown in Fig. 3.

**Table 3:** Biases for scar volume using PSIR-based imaging relative to MIR-based imaging. Results are presented as a percent of LV volume according to Bland-Altman analysis for different etiologies and thresholding techniques

Etiology	ICM				NICM		
Thresholding technique	STRM			FWHM	STRM		
	2SD	3SD	5SD	FWHM	2SD	3SD	5SD
Bias	−3.27	−3.89	−4.87	+6.89	−9.71	−7.41	−4.08

*ICM* ischemic cardiomyopathy; *NICM* non-ischemic cardiomyopathy; *STRM* signal threshold versus reference myocardium; *FWHM* full-width at half-maximum

### PSIR versus MIR scar volumes – FWHM-based analysis

**Fig. 4:**
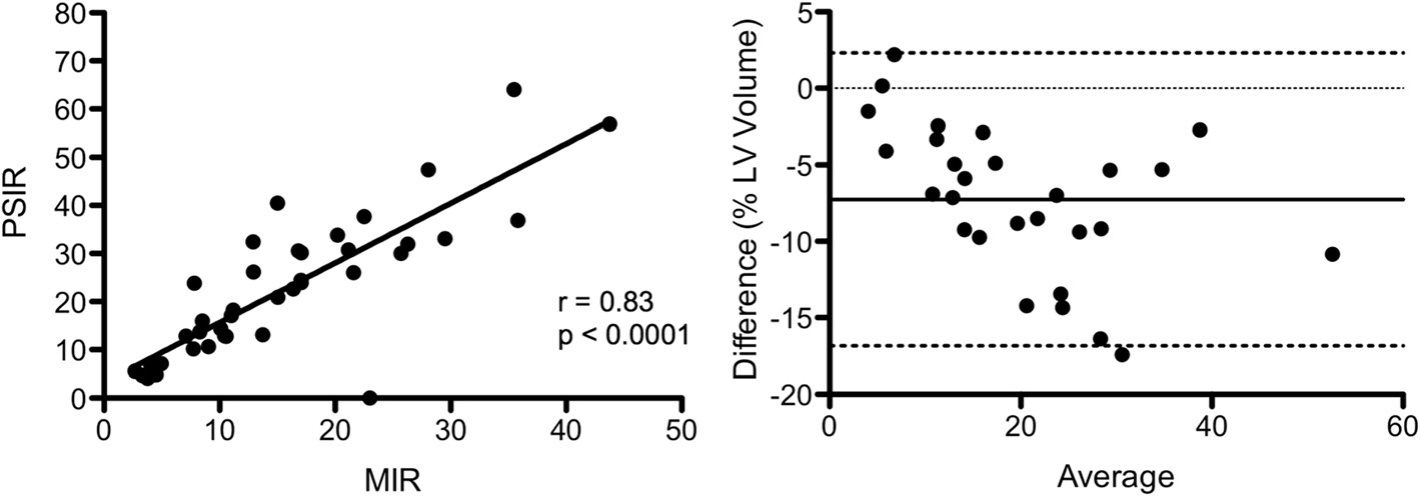
Linear regression and Bland-Altman analyses comparing myocardial late enhancement volume using MIR and PSIR for FWHM-based analysis. Late enhancement is reported as percent of total LV myocardial volume at 2, 3, and 5 SD from the mean signal intensity of normal reference myocardium. **Left:** Linear regression analysis. Values indicate the mean difference of MIR-based scar volume quantification relative to PSIR. **Right:** Bland-Altman analysis. Long dashed lines represent bias, dotted lines represent 95 % confidence intervals

Among those with ischemic injury, scar signal quantification was repeated using the FWHM technique, as previously described [20]. Linear regression analysis demonstrated a good correlation between MIR and PSIR derived total scar volumes (r = 0.83, *p* < 0.0001). Bland-Altman analysis demonstrated a systematic bias between MIR and PSIR derived measurements of total myocardial scar volume. However, in contrast to STRM-based imaging, the mean bias demonstrated a reduction in total scar volume estimates using MIR-based images versus PSIR-based images, and was estimated to be 6.9 % of the LV mass (Fig. 4).

### PSIR versus MIR scar volumes—background myocardial signal

The influence of PSIR reconstruction on measurable background myocardial signal was assessed among 40 control patients. As this population lacks visible scar only STRM-based analysis is applicable. Measurable signal was appreciated in all patients and was inversely related to the threshold employed. Using MIR-based images the mean total “scar” volume was 13.6 ± 5.6 %, 6.1 ± 3.1 % and 1.3 ± 0.9 % of the LV at ≥2SD, ≥3SD and ≥5SD thresholds, respectively (Table 2). Correlation coefficients for ≥2SD, ≥3SD and ≥5SD thresholds were 0.33, 0.58, and 0.66, respectively (Fig. 2). Bland-Altman analysis showed a systematic bias with PSIR images producing a reduction in mean total “scar” volume of 4.4 %, 2.6 %, and 0.7 % of the LV mass at ≥2SD, ≥3SD and ≥5SD thresholds, respectively (Fig. 3).

## Discussion

Myocardial scar volume quantification is an established investigative and emerging clinical tool for the prediction of adverse clinical outcomes and therapeutic response in patients with ischemic and non-ischemic cardiomyopathy [3–8]. To date such evidence is based upon signal threshold analysis of conventional, MIR-based LGE CMR. However, rapid and widespread clinical adoption of PSIR-based LGE sequences is being realized following recent introduction by a majority of hardware vendors. The current study identifies that signal-threshold based scar volume quantification should not be considered equivalent between these techniques, and may lead to clinically relevant differences in scar volume estimations dependent upon the analysis technique used, signal threshold chosen or type of scar evaluated.

Our results are at odd with previous studies comparing MIR and PSIR-based scar signal analysis by manual tracing of LGE. In these studies, volume of LGE did not vary significantly between techniques [15–18]. This may be explained by the fact that the marked signal profile change secondary to phase correction is largely un-recognized by visual interpretation as adjustment of window-level settings automatically re-establishes similar voxel intensities for the myocardium.

To our knowledge, only one previous study used a signal threshold technique [19] for quantification of LGE volume. Although the difference was not significant, LGE volume quantification by MIR-based scar signal analysis was higher than PSIR values. This study, however had fewer patients and did not assess FWHM-based analysis.

A number of factors likely contribute in a complex manner to produce the biases identified in this study between PSIR versus MIR-based scar signal analysis. First, it is anticipated that T1 relaxation properties of the normal myocardium vary to some degree throughout the heart’s volume as a results of varying tissue composition and with B1-inhomogeneity [23]. Further, T1 will dynamically change throughout the course of imaging as gadolinium washes out of the myocardium [24, 25]. Despite careful optimization of the TI time to provide satisfactory nulling in a majority of the myocardium, voxel heterogeneity in T1 relaxation cannot be accommodated for using a single TI time prescribed over the entire myocardium at the onset of imaging. While potentially not appreciated by visual inspection, inadequately nulled voxels will receive higher signal using MIR-based reconstruction and, if exceeding a threshold value dictated by referenced tissue, be labeled as scar.

Second, the distribution of noise within magnitude and phase-reconstructed images is substantially different. For example, MIR images are typically reconstructed using multi-channel sum-of-squares, a technique that produces signal in low SNR regions (i.e. nulled myocardium) that roughly follows a centric chi-squared (Rician) distribution, which is positively skewed [25]. This creates the potential for voxels in nulled (ie: normal) myocardium to be misclassified as “scar”, a finding that was evident in the control population of the current study. In contrast, PSIR images preserve the polarity information in the data such that the myocardium maintains intermediate signal intensity. The normal myocardium is therefore “grey” rather than “nulled” (although this is infrequently realized when viewing window-level adjusted images). Therefore, noise signal is roughly normally distributed (non-skewed distribution) within normal myocardium using PSIR imaging [15] and therefore is less likely to mislabel noise signal as scar.

An interesting observation was that FWHM scar analysis produced an opposite bias for scar signal analysis than for STRM. This can be theoretically explained by considering the net influence of signal alterations for reference versus target tissues. The FWHM approach adopts the maximal signal intensity of scar as its reference signal with 50 % of this signal used as the threshold above which scar will be defined. The maximal signal within scar (and hence, the scar signal threshold) varies modestly when phase reconstruction is applied given that this high signal is Gaussian in distribution. However, the lower signal ranges of more peripheral scar signal are expected to be shifted more substantially. This will be intermediate to that experienced by normal or “nulled” myocardium that is re-set from approximately zero signal (ie: nulled) to a mid-range signal intensity (ie: grey) [15]. Overall, this will lead to a higher likelihood of intermediate signal voxels to be re-classified from “normal” to “scar” using the FWHM approach.

Overall, while in practice the situation is incrementally more complex with respect to changes in noise statistics, filtering of the reference image and combination of B1 data from multiple coils [14], the net result is that phase reconstruction influences a host of variables that may alter image signal profiles sufficient that threshold-based quantification may yield dissimilar results versus that performed from matched magnitude-reconstructed images.

### Clinical implications

The primary implications of these findings relate to the planning of future multi-center research initiatives that may have a heterogeneous adoption of MIR and PSIR-based imaging among recruiting sites. It should be realized that images obtained using these alternate approaches might yield a range of differences in scar volume despite standardized core-laboratory analysis that could reach clinically relevant levels. For example, among those with non-ischemic injury, scar volumes differences ranging up to 9.7 % may be realized using STRM-based analysis. For those with ischemic injury differences up to 4.9 % by STRM analysis and 6.9 % by FWHM may be realized. Practical solutions to this might include the collection of TI-optimized MIR images in concert with PSIR images (as performed in the current study), or the correction of scar volumes by the described bias values.

### Study limitations

This study was performed using a 3 Tesla Siemens MR system coupled with a 32-channel coil. Accordingly, generalizability of these findings to other system configurations cannot be recommended. Further, the dose and relaxivity of different gadolinium based contrast agents may contribute to alterations in signal based quantification and was not tested in this study.

## Conclusions

Phase reconstruction of LGE images provides substantial practical benefits to clinical workflow and has the potential to improve image quality at less experienced centers. We however identify systematic bias that may be introduced for the performance of signal-threshold based scar quantification dependent upon both etiology and the scar threshold technique employed. These findings warrant consideration when planning multi-center research initiatives and for the translation of scar quantification into clinical practice.
